# Association between stress hyperglycemia ratio (SHR) and long-term mortality in patients with ischemic stroke: a retrospective cohort study

**DOI:** 10.1186/s12933-025-02730-8

**Published:** 2025-04-25

**Authors:** Man Huang, Wan Wang, Dong-mei Ren, Yan-qing Chen, Ying Li, Yan Li, Wu-lin Li, Fei Wang

**Affiliations:** 1 Department of Nursing, Jiading District Central Hospital Affiliated Shanghai University of Medicine & Health Sciences, Shanghai, China; 2 Department of Neurology, Jiading District Central Hospital Affiliated Shanghai University of Medicine & Health Sciences, Shanghai, China; 3 Shanghai Key Laboratory of Molecular Imaging, Department of Emergency and Critical Care Medicine, Jiading District Central Hospital Affiliated Shanghai University of Medicine & Health Sciences, No.1, Chengbei Rd, Jiading District, Shanghai, China

**Keywords:** Ischemic stroke, Stress hyperglycemia ratio, Long-term mortality, Predictive, Cohort study

## Abstract

**Background:**

The stress hyperglycemia ratio (SHR) at the time of admission has been linked to short-term adverse outcomes in patients suffering from ischemic stroke (IS). However, the connection between SHR and long-term mortality in cases of IS has yet to be investigated. This study aimed to elucidate the connection between SHR and long-term mortality in IS patients, while also investigating the impact of stratification status on this relationship.

**Methods:**

Data regarding IS patients were extracted from our medical institution’s undisclosed internal stroke database, spanning from January 2016 to December 2023. Participants were classified into three groups according to the tertiles of continuous SHR. The primary outcome centered on all-cause mortality over a six-year period, whereas the secondary outcome focused on in-hospital all-cause mortality. Cox regression analysis and Kaplan-Meier curves were utilized to assess the connection between SHR and mortality rates. To further investigate the nature of this relationship, a restricted cubic spline (RCS) analysis was performed to determine its linearity, and an iterative algorithm was employed to pinpoint the inflection points. Variations among the strata were depicted in a subgroup forest plot. The prognostic ability of SHR concerning mortality risk was illustrated through receiver operating characteristic (ROC) curves.

**Results:**

Among the 4330 participants, the mean age was 69.3 ± 13.4 years, with 2805 individuals (64.8%) identified as male. SHR was linked to a heightened risk of all-cause mortality at the six-year follow-up (HR 1.605, 95% CI 1.099–2.345) and during hospitalization (HR 3.698, 95% CI 1.950–7.014) (*P* < 0.05). The RCS analysis uncovered a “U-shaped” nonlinear relationship between SHR and six-year all-cause mortality. Subgroup analyses revealed that, among the non-diabetic cohort, patients devoid of atrial fibrillation, and those who had not undergone endovascular treatment, both low and high SHR significantly elevated the six-year mortality risk compared to moderate SHR.

**Conclusion:**

This study revealed that SHR is correlated with a heightened risk of six-year and in-hospital all-cause mortality in IS patients. A U-shaped nonlinear association is observed between SHR and six-year all-cause mortality. Therefore, SHR could potentially act as a significant predictor for adverse long-term outcomes in IS patients, thereby facilitating clinical decision-making and risk evaluation.

**Graphical abstract:**

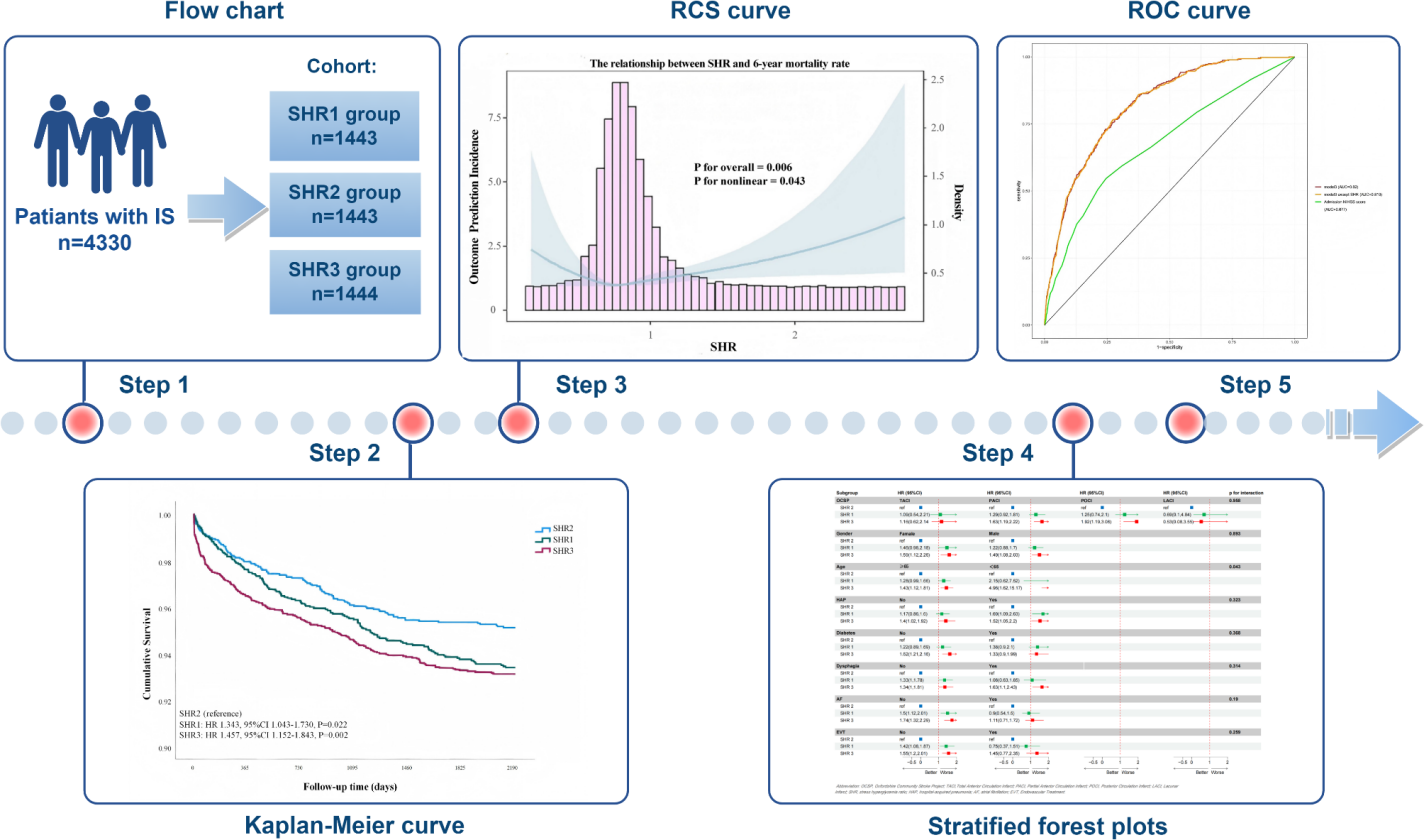

**Supplementary Information:**

The online version contains supplementary material available at 10.1186/s12933-025-02730-8.

## Background

Stroke is a sudden neurological condition resulting from the interruption of cerebral blood flow, leading to either localized or widespread neurological deficits, with ischemic strokes constituting approximately 84.4% of all cases [1]. Over the last six years, mortality rates associated with strokes have experienced a downward trend; however, stroke continues to be the second leading cause of death globally and remains the primary cause of acquired long-term disability, contributing to an increasing annual economic burden on a worldwide scale [2]. Annually, more than 13.7 million strokes occur globally, culminating in approximately 5.5 million fatalities each year [3]. Researchers emphasize the critical importance of early detection and prediction of factors associated with mortality [4], and studies have shown a significant correlation between the triglyceride-glucose index and all-cause mortality among critically ill ischemic stroke (IS) patients in both hospital and intensive care unit settings [5–6]. Furthermore, factors influencing mortality in IS include low arterial blood partial pressure of oxygen, atrial fibrillation (AF), physical inactivity, an initial thrombolysis in cerebral infarction score of 1, a modified Rankin scale (mRS) score of 5, neutrophil percentage, the initial national institute of health stroke scale (NIHSS), anemia, malnutrition, and initial blood glucose levels [7, 8, 9, 10].

Further exploration into the relationship between fasting blood glucose and a variety of diseases has revealed a J-shaped dose–response correlation between fasting blood glucose levels and both overall mortality and cancer mortality [11]. Fasting plasma glucose acts as an independent prognostic indicator for negative outcomes at 90 days post-stroke in patients with acute ischemic stroke (AIS). When fasting blood glucose reaches or surpasses 5.5 mmol/L, the probability of adverse outcomes within 90 days significantly increases [12]. However, assessing emergent hyperglycemia is challenging due to fluctuating baseline blood glucose levels, which can lead to inaccurate evaluations of emergent levels [13]. In 2015, Roberts et al. proposed the stress hyperglycemia ratio (SHR) to enable a more precise assessment by considering individual differences in glucose metabolism [14]. Numerous researchers have investigated the association between the prognosis of IS patients and the SHR, demonstrating that the SHR, derived from glucose/glycated hemoglobin (HbA1c), is correlated with a reduced likelihood of favorable functional outcomes within 90 days for individuals experiencing acute large vessel occlusion stroke [15]. The SHR is intricately linked to an increased risk of negative outcomes and mortality in AIS patients, characterized by a nonlinear dose–response relationship, implying that the SHR could potentially serve as a novel predictor of poor mortality and prognosis in these individuals [16, 17, 18]. While existing research provides relevant evidence that the SHR can anticipate mortality in AIS patients, the duration of follow-up for these individuals is limited, leaving their long-term survival status unexplored. This study aims to investigate the connection between SHR and long-term all-cause mortality in IS patients, building on the findings of previous scholars, while also examining the stratifying factors that may influence this relationship.

## Methods

### Data sources

Data were obtained from an undisclosed internal stroke database.

### Study design and population

This study was designed as a retrospective cohort study. The design process of this study is shown in, Graphical Abstract.

A total of 4330 patients diagnosed with IS were admitted and hospitalized at the Jiading District Central Hospital, affiliated Shanghai University of Medicine & Health Sciences, from January 2016 to December 2023. All individuals included in this analysis met the established inclusion and exclusion criteria. The diagnostic criteria for IS in our study were based on the guidelines set forth by Chinese guidelines for diagnosis and treatment of AIS (version 2014 [19] was applied before 2018 and version 2018 [20] was applied after that). Diagnostic criteria for IS [19, 20] include (1) Sudden onset; (2) Presence of focal neurological deficits, such as unilateral weakness or numbness in the face or limbs, language impairments, among others, which may manifest as severe overall neurological dysfunction; (3) Imaging studies revealing definitive lesions or symptoms/signs persisting for over 24 h; (4) Exclusion of non-vascular etiologies; (5) Cerebral CT/MRI conducted to eliminate the possibility of cerebral hemorrhage. The final diagnosis was corroborated by experienced neurologists who reviewed the brain imaging results and clinical presentations of the patients. This multi-faceted approach ensured a robust and accurate diagnosis of IS.

The participants were divided into three separate cohorts according to the tertiles of the continuous SHR.

Inclusion and exclusion criteria.

The inclusion criteria were as follows: (1) IS patients diagnosed by brain imaging and met the diagnostic criteria; (2) No cerebral hemorrhage was found in the first head CT scan after admission; (3) the first onset; (4) complete clinical data.

Exclusion criteria were as follows: (1) a definite diagnosis of community-acquired pneumonia; (2) fever or active infection within 2 weeks before admission; (3) long-term use of immunomodulatory drugs or glucocorticoids; (4) use of antibiotics before admission; (5) complicated with malignant tumors; (6) combined with hematological diseases; (7) combined with autoimmune diseases; (8) the data provider has speech communication disorder, consciousness disorder, mental disorders; (9) incomplete data.

This study was approved by the Ethics Committee of Shanghai Jiading District Central Hospital in accordance with the Declaration of Helsinki. Informed consent was waived based on the design of this study.

### Data extraction

Demographic data: gender, age, body mass index (BMI, kg/m2) and waist circumference.

Personal history: Smoking status, the Oxfordshire community stroke project (OCSP), the trial of org 10,172 in acute stroke treatment (TOAST), and time from stroke onset to hospitalization.

Medical histories: prior cerebral infarction, transient ischemic attack, myocardial infarction, hypertension, diabetes mellitus, atrial fibrillation, hyperlipidemia, cerebral hemorrhage, dementia, mental health disorder, chronic obstructive pulmonary disease, spontaneous intracerebral hemorrhage, and a family history of stroke, alongside heart valve replacement surgery. All medical histories were meticulously gathered within 8 h post-admission in accordance with specialized diagnostic guidelines for diseases.

Severity of the disease: The mRS score before onset, the admission NIHSS score, dysphagia status, mean arterial pressure (MAP) and pulse rate were recorded. The data were collected within 24 h after admission. The mRS score and NIHSS score at admission represent the metrics recorded prior to treatment and pharmaceutical intervention. The values for MAP and heart rate are calculated as averages over a 24-hour timeframe.

Treatment methods: Hypoglycemic agents, antiplatelet therapy, anticoagulant drugs, antihypertensive medications, lipid-lowering treatments, Endovascular Treatment (EVT), intravenous thrombolysis utilizing alteplase, and mechanical thrombectomy.

Laboratory indicators: low-density lipoprotein (LDL), homocysteine (Hcy), HbA1c, admission blood glucose (ABG), serum creatinine (SCr), blood urea nitrogen (BUN), uric acid (UA), international normalized ratio (INR), SHR (calculated according to ABG and HbA1c).

The clinical prognosis data included length of hospital stay, hospitalization cost, hospitalization drug cost, hospital-acquired pneumonia (HAP) occurrence during hospitalization, in-hospital mortality, and six-year survival status. The death information of patients, based on their unique identification number, is tracked in the ‘Wanda Death’ system, a comprehensive database designed to accurately register and query the mortality data of residents. All results are from the initial assessment conducted within 24 h of admission to the neurology unit and based on treatments performed during the main period. The data has been meticulously processed and systematically organized within an Excel database.

### Definition of data

The body mass index (BMI) was calculated as weight (kg) divided by the square of height (m). The SHR was determined using the following formula: SHR = admission blood glucose (ABG) (mmol/L)/[1.59×HbA1c (%) − 2.59] [14]. Diabetes was defined as a history of diabetes or HbA1c > 6.5%. Pre-DM was defined as patients without a history of diabetes but with HbA1c levels between 5.7 and 6.4%. Normoglycemia (NGR) was identified in patients without a history of diabetes or with HbA1c ≤ 5.7% [21].

### Study outcomes

The primary outcome of this study was 6-year mortality and the secondary outcome was in-hospital mortality.

### Statistical methods

As the current study is a retrospective analysis, no sample size calculations were conducted. Variables with a missing data rate exceeding 20% were excluded, and for those with less than a missing data rate below 20%, multiple imputation was employed. Medical records were divided into three groups based on the baseline SHR tertiles which was continuous variable data, with the second tertile group (SHR2 group) as the reference group. The continuous variables were tested for normality. Wilcoxon rank sum test was used for non-normally distributed data, and the results were expressed as the median of interquartile range (IQR). Categorical variables were analyzed with the use of the chi-square test or fisher’s exact test and are expressed as absolute numbers and percentages. To analyze the association between the SHR and the risk of the primary and secondary outcomes, hazard ratios (HRs) and 95% confidence intervals were calculated using multivariate Cox proportional hazards regression models. Model 1 did not adjust for any covariates, model 2, Factors were derived from a multivariate Cox regression analysis of factors after intentional single factors in supplement Table 2. model 3, based on model 2 factors and clinical features and treatment to adjust. Kaplan-Meier survival analysis was used to assess the risk of mortality in 6-year and in-hospital. In addition, the SHR was also analyzed, and restricted cubic splines (RCS) were used to clarify the association of dose-response with the risk of primary and secondary outcomes. If the association was nonlinear, a recursive algorithm was used to determine the inflection points in the 6-year and in-hospital mortality, a two-stage Cox proportional hazards model was applied to analyze the threshold effect of SHR on mortality in IS patients on both sides of the inflection-point. Furthermore, stratified analyses were conducted according to OCSP classification, gender, age, HAP, diabetes, AF, dysphagia and EVT. To evaluate the improvement in predictive accuracy when incorporating SHR into the existing prognostic scoring system, we employed the receiver operating characteristic (ROC) curve, quantifying the enhancement via the area under curve (AUC). Statistical analyses were performed using SPSS26.0 software and R software, and the significance level was set at *P* < 0.05.

**Table 1:** Baseline characteristics comparison between the three SHR groups

Factors	Total (*n* = 4330)	SHR 1(*n*) (0.175-0759, *n* = 1443)	SHR 2 (*n* ) (0.759–0.885, *n* = 1443)	SHR 3 (*n* ) (0.885–2.760, *n* = 1444)	*P* Value
Demographic data					
Male [n (%)]	2805 (64.78)	985 (68.26)	942 (65.28)	878 (60.80)	< 0.001
Age (Mean ± SD, years)	69.28 ± 13.41	69.30 ± 12.98	68.56 ± 13.42	69.99 ± 13.80	0.016
BMI (Mean ± SD)	24.46 ± 2.98	24.32 ± 3.12	24.57 ± 2.89	24.50 ± 2.92	0.061
Waist circumference (Mean ± SD, cm)	84.18 ± 8.31	83.91 ± 8.25	84.53 ± 8.46	84.09 ± 8.22	0.139
OCSP [n (%)]					0.26
TACI [n (%)]	421 (9.72)	132 (9.15)	144 (9.98)	145 (10.04)	
PACI [n (%)]	2605 (60.16)	884 (61.26)	833 (57.73)	888 (61.50)	
POCI [n (%)]	1110 (25.64)	362 (25.09)	392 (27.17)	356 (24.65)	
LACI [n (%)]	194 (4.48)	65 (4.50)	74 (5.13)	55 (3.81)	
TOAST [n (%)]					0.161
Large-artery atherosclerosis [n (%)]	1686 (38.94)	583 (40.40)	535 (37.08)	568 (39.34)	
Cardio embolism [n (%)]	473 (10.92)	141 (9.77)	152 (10.53)	180 (12.47)	
Small-vessel occlusion [n (%)]	2025 (46.77)	665 (46.08)	710 (49.20)	650 (45.01)	
Stroke of other determined etiology [n (%)]	55 (1.27)	21 (1.46)	19 (1.32)	15 (1.04)	
Stroke of undetermined etiology [n (%)]	91 (2.10)	33 (2.29)	27 (1.87)	31 (2.15)	
Smoke [n (%)]					< 0.001
Never smoked [n (%)]	3074 (70.99)	920 (63.76)	1058 (73.32)	1096 (75.90)	
Used to smoke [n (%)]	208 (4.80)	72 (4.99)	68 (4.71)	68 (4.71)	
Still smoking [n (%)]	1048 (24.20)	451 (31.25)	317 (21.97)	280 (19.39)	
Duration from symptom onset to hospital [M (25%,75%), hours]	8.0 (2.1, 30.0)	9.0(2.2,33.8)	8.2 (2.1,27.9)	6. 9(1.9,29.1)	0.018
Past medical history [n (%)] [n (%)]					
Prior cerebral infarction [n (%)]	797 (18.41)	270 (18.71)	257 (17.81)	270 (18.70)	0.774
Transient ischemic attack [n (%)]	20 (0.46)	5 (0.35)	5 (0.35)	10 (0.69)	0.286
Myocardial infarction [n (%)]	26 (0.60)	8 (0.55)	3 (0.21)	15 (1.04)	0.015
Hypertension [n (%)]	3110 (71.82)	987 (68.40)	1058 (73.32)	1065 (73.75)	0.002
Diabetes mellitus [n (%)]	1314 (30.35)	487 (33.75)	342 (23.70)	485 (33.59)	< 0.001
Atrial fibrillation [n (%)]	536 (12.38)	170 (11.78)	147 (10.19)	219 (15.17)	< 0.001
Hyperlipidemia [n (%)]	27 (0.62)	10 (0.69)	9 (0.62)	8 (0.55)	0.826
Cerebral hemorrhage [n (%)]	104 (2.40)	39 (2.70)	32 (2.22)	33 (2.29)	0.654
Dementia [n (%)]	41 (0.95)	19 (1.32)	13 (0.90)	9 (0.62)	0.153
Mental health disorder [n (%)]	21 (0.48)	3 (0.21)	10 (0.69)	8 (0.55)	0.155
Chronic obstructive Pulmonary disease [n (%)]	72 (1.66)	28 (1.94)	23 (1.59)	21 (1.45)	0.575
Spontaneous intracerebral hemorrhage [n (%)]	54 (1.25)	20 (1.39)	12 (0.83)	22 (1.52)	0.208
Family history of stroke [n (%)]	15 (0.35)	7 (0.49)	5 (0.35)	3 (0.21)	0.4
Heart valve replacement surgery [n (%)]	7 (0.16)	4 (0.28)	1 (0.07)	2 (0.14)	0.423
The severity of the disease					
Pre-morbidity mRS score [M (IQR)]	2.00 (1.00, 2.00)	2.00 (1.00,2.00)	1.00 (1.00,2.00)	1.00 (1.00,2.00)	0.002
Admission NIHSS score [M (IQR)]	2.00 (1.00, 5.00)	2.00 (1.00,4.00)	2.00 (1.00,5.00)	3.00 (1.00,8.00)	< 0.001
Dysphagia [n (%)]	489 (11.44)	80 (5.60)	122 (8.59)	287 (20.15)	< 0.001
MAP (Mean ± SD, mmHg)	105.94 ± 14.25	104.42 ± 13.00	106.09 ± 14.24	107.33 ± 15.27	< 0.001
Pulse (Mean ± SD, Times/minute)	77.76 ± 14.36	76.48 ± 13.53	76.90 ± 13.60	79.88 ± 15.61	< 0.001
Therapeutic regimen					
Hypoglycemic agents [n (%)]	1477 (34.11)	548 (37.98)	403 (27.93)	526 (36.43)	< 0.001
Antiplatelet drug therapy [n (%)]	4163 (96.14)	1417 (98.20)	1395 (96.67)	1351 (93.56)	< 0.001
anticoagulant drugs [n (%)]	115 (2.66)	42 (2.91)	26 (1.80)	47 (3.25)	0.04
antihypertensive medications [n (%)]	3315 (76.56)	1071 (74.22)	1142 (79.14)	1102 (76.32)	0.007
lipid-lowering treatments [n (%)]	4131 (95.40)	1410 (97.71)	1394 (96.60)	1327 (91.90)	< 0.001
Endovascular Treatment [n (%)]	671 (15.50)	193 (13.37)	226 (15.66)	252 (17.45)	0.01
Intravenous thrombolysis utilizing alteplase [n (%)]	650 (15.01)	189 (13.10)	220 (15.25)	241 (16.69)	0.025
Mechanical thrombectomy [n (%)]	62 (1.50)	16 (1.16)	13 (0.95)	33 (2.39)	0.003
Laboratory parameters					
LDL (M (IQR), mmol/L)	2.66 (2.05, 3.30)	2.63 (2.03,3.26)	2.68 (2.05,3.31)	2.65 (2.07,3.35)	0.294
Hcy [M (IQR), umol/L]	14.80 (11.40, 20.20)	14.80 (11.40,20.17)	14.90 (11.50,20.02)	14.60 (11.30,20.40)	0.907
HbA1c (Mean ± SD)	6.67 ± 1.73	7.04 ± 1.82	6.47 ± 1.54	6.49 ± 1.76	< 0.001
ABG (Mean ± SD, mmol/L)	6.70 ± 2.69	5.68 ± 1.76	6.31 ± 2.05	8.11 ± 3.36	< 0.001
SCr [M (IQR), umol/L]	72.75 (61.61, 86.29)	74.55 (63.36,88.05)	72.00 (61.18,85.20)	71.26 (60.28,85.20)	< 0.001
BUN [M (IQR), mmol/L]	5.10 (4.20, 6.20)	5.00 (4.20,6.10)	5.00 (4.10,6.10)	5.20 (4.20,6.70)	< 0.001
UA (Mean ± SD, umol/L)	330.47 ± 102.11	332.32 ± 102.48	330.12 ± 99.39	328.96 ± 104.44	0.669
INR (Mean ± SD)	0.95 ± 0.15	0.95 ± 0.13	0.95 ± 0.16	0.96 ± 0.16	0.322
SHR (Mean ± SD)	0.85 ± 0.20	0.67 ± 0.08	0.82 ± 0.04	1.05 ± 0.20	< 0.001
Outcomes					
Length of hospitalization [M (IQR), days]	10.00 (8.00, 13.00)	10.00 (8.00,12.00)	10.00 (8.00,13.00)	11.00 (8.00,14.00)	< 0.001
Total hospitalization expenses [M (IQR), Thousand yuan]	14.7(8.6)	14.0(7.2)	14.6(8.3)	15.8(10.4)	< 0.001
Total hospitalization drug expenses [M (IQR), Thousand yuan]	7.1(6.0)	6.5(5.2)	7.0(6.0)	7.9(7.2)	< 0.001
HAP [n (%)]	804 (18.57)	173 (11.99)	221 (15.32)	410 (28.39)	< 0.001
Died in hospital [n (%)]	64(1.5)	7(0.5)	10(0.7)	47(3.3)	< 0.001
Died in 6 years [n (%)]	481 (11.11)	143 (9.91)	118 (8.18)	220 (15.24)	< 0.001

ABG, admission blood glucose; BMI, body mass index; BUN, blood urea nitrogen; Hcy, homocysteine; HbA1c, glycated hemoglobin; HAP, hospital-acquired pneumonia; INR, international normalized ratio; LACI, lacunar infarct; LDL, low-density lipoprotein; MAP, mean arterial pressure; mRS, modified rankin scale; NIHSS, national institute of health stroke scale; OCSP, oxfordshire community stroke project; POCI, posterior circulation infarct; PACI, partial anterior circulation infarct; SHR, stress hyperglycemia ratio; SCr, serum creatinine; TACI, total anterior circulation infarct; TOAST, trial of org 10172 in acutes troke treatment; UA, uric acid.

## Results

### Descriptive analysis

**Fig. 1:**
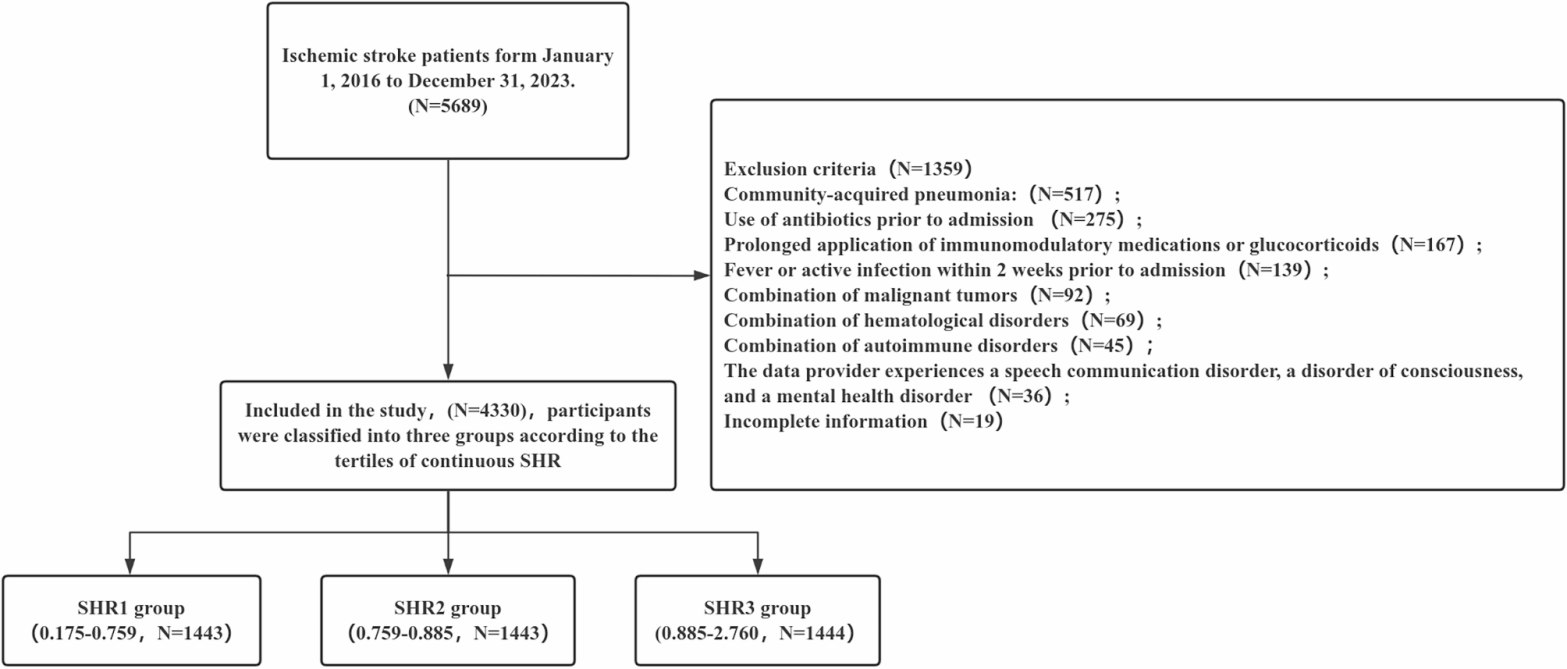
Flow chart

A cumulative total of 5689 cases were systematically gathered, with 1359 cases omitted, resulting in 4330 cases ultimately incorporated into the analysis. Among these participants, males constituted 64.8% (2805/4330), and the mean age was 69.3 ± 13.4 years. The patients were categorized into three distinct groups based on the tertiles of SHR at the time of admission: SHR1 group (0.175–0.759, *n* = 1443), SHR2 group (0.759–0.885, *n* = 1443), and SHR3 group (0.885–2.760, *n* = 1444). Refer to Fig. 1.

### Baseline characteristics of study individuals

The baseline characteristics of the three groups were presented in Table 1. Participants in the SHR3 group were significantly older, predominantly female, and exhibited a higher prevalence of myocardial infarction, hypertension, atrial fibrillation, and dysphagia. Additionally, parameters such as the admission NIHSS score, MAP, pulse rate, ABG, SHR, and BUN were markedly elevated. This group also displayed increased rates of anticoagulant therapy utilization, EVT, rtPA thrombolysis, mechanical thrombectomy, hospitalization costs, hospitalization drug costs, incidence of HAP, Scr levels, and a shortened time from stroke onset to hospitalization, along with a reduced reliance on antiplatelet and lipid-lowering agents. The SHR3 cohort revealed significantly higher in-hospital and six-year mortality rates compared to the other groups, with six-year mortality rates documented at 15.2% versus 8.2% versus 9.9% (*P* < 0.001) and in-hospital mortality rates at 3.3% versus 0.7% versus 0.5% (*P* < 0.001).

**Table 2:** Multivariate Cox regression analysis of six-year and in-hospital mortality among IS patients

	Model1		Model2		Model3	
	HR (95%CI)	*P*	HR (95%CI)	*P*	HR (95%CI)	*P*
*Six-year mortality*						
SHR as a continuous variable	3.690 (2.630–5.179)	< 0.001	1.642 (1.132–2.380)	0.009	1.605 (1.099–2.345)	0.014
SHR divided into tertiles						
SHR2 group	1.0 (reference)		1.0 (reference)		1.0 (reference)	
SHR1 group	1.218 (0.954–1.554)	0.113	1.275 (0.996–1.633)	0.054	1.343 (1.043–1.730)	0.022
SHR3 group	1.959 (1.566–2.450)	< 0.001	1.417 (1.126–1.784)	0.003	1.457 (1.152–1.843)	0.002
P for trend	< 0.001		< 0.001		< 0.001	
*In-hospital mortality*						
SHR as a continuous variable	7.940 (4.461–14.131)	< 0.001	3.839 (2.013–7.319)	< 0.001	3.698 (1.950–7.014)	< 0.001
SHR divided into tertiles						
SHR2 group	1.0 (reference)		1.0 (reference)		1.0 (reference)	
SHR1 group	0.725 (0.276–1.905)	0.514	0.946 (0.356–2.511)	0.911	0.875 (0.330–2.321)	0.789
SHR3 group	3.956 (1.989–7.868)	< 0.001	2.874 (1.438–5.742)	0.003	2.569 (1.281–5.150)	0.008
P for trend	< 0.001		< 0.001		< 0.001	

In a multivariate COX regression of 6-year mortality

Model 1: no adjustment

Model 2: adjusted for age, gender, antiplatelet drug therapy, lipid-lowering treatments, HAP, prior cerebral infarction, dysphagia, diabetes mellitus (Factors were derived from a multivariate COX regression analysis of factors after intentional single factors in supplement Table 2)

Model 3: adjusted for age, gender, antiplatelet drug therapy, lipid-lowering treatments, HAP, prior cerebral infarction, dysphagia, diabetes mellitus, admission NIHSS score, hypertension, myocardial infarction, anticoagulant drugs, atrial fibrillation, hyperlipidemia, chronic obstructive pulmonary disease, dementia (Based on Model 2 factors and clinical features and treatment)

In multivariate COX regression of in-hospital mortality

Model 1: no adjustment

Model 2: adjusted for age, gender, antiplatelet drug therapy, OCSP, TOAST, SCr, admission NIHSS score (Factors were derived from a multivariate COX regression analysis of factors after intentional single factors in supplement Table 2)

Model 3: adjusted for age, gender, antiplatelet drug therapy, OCSP, TOAST, SCr, admission NIHSS score, HAP, hypertension, diabetes mellitus, myocardial infarction, prior cerebral infarction, anticoagulant drugs, atrial fibrillation, hyperlipidemia, chronic obstructive pulmonary disease, dementia (Based on Model 2 factors and clinical features and treatment)

HAP, hospital-acquired pneumonia; NIHSS, national institute of health stroke scale; OCSP, oxfordshire community stroke project; SHR, stress hyperglycemia ratio; SCr, serum creatinine; LDL, low-density lipoprotein; TOAST, trial of org 10172 in acute stroke treatment

### Relationship between the SHR and clinical outcomes in IS patients

The multivariate Cox regression analysis of factors (supplement Table 3) showing significant differences indicated that antiplatelet and lipid-lowering therapies acted as protective agents against the risk of mortality over a six-year period. In contrast, age, diabetes mellitus, dysphagia, prior cerebral infarction, and HAP surfaced as risk factors for six-year mortality. Even after accounting for these variables, SHR continued to demonstrate a significant correlation with the six-year mortality. The hazard ratios (HRs) and 95% confidence intervals (CIs) for all-cause mortality over a six-year period in the SHR1 and SHR3 groups, relative to the SHR2 group, were 1.275 (0.996–1.633) and 1.417 (1.126–1.784), respectively. The HRs for six-year all-cause mortality associated with the SHR were 1.642 (1.132–2.380), *P* < 0.001.

**Table 3:** Analysis of threshold effects of SHR on 6-year and in-hospital mortality in IS patients

	HR (95%CI), *P* value
*Six-year mortality*	
Model 1 Fitting model by standard linear regression	3.69(2.63–5.179), < 0.001
Model 2 Fitting model by two-piecewise linear regression	
Inflection point	0.76
< 0.76	0.281(0.067–1.181), 0.083
> 0.76	4.877(3.443–6.907), < 0.001
P for likelihood ratio test	< 0.001
*In-hospital mortality*	
Model 1 Fitting model by standard linear regression	3.517(1.802–6.863), < 0.001
Model 2 Fitting model by two-piecewise linear regression	
Inflection point	1.165
< 1.165	31.533(5.571-178.476), < 0.001
> 1.165	1.102(0.319–3.802), 0.878
P for likelihood ratio test	0.006

After adjusting for variables such as age, gender, antiplatelet drug therapy, lipid-lowering treatments, HAP, prior cerebral infarction, dysphagia, diabetes mellitus, admission NIHSS score, hypertension, myocardial infarction, anticoagulant drugs, atrial fibrillation, hyperlipidemia, chronic obstructive pulmonary disease, dementia (Model 3 in Table 2), the hazard ratios (HRs) and 95% confidence intervals (CIs) for all-cause mortality over six years in the SHR1 and SHR3 groups, compared to the SHR2 group, were 1.343 (1.043–1.730) and 1.457 (1.152–1.843). The HRs for six-year all-cause mortality based on the SHR were 1.605 (1.099–2.345), *P* < 0.01.

The multivariate Cox regression analysis indicated that SHR constituted an independent risk factor for in-hospital mortality. When comparing the SHR2 group, the SHR1 group showed no significant difference in in-hospital mortality rates, while the SHR3 group exhibited a notable increase in the risk of in-hospital mortality, as delineated in Table 2.

### Survival analysis

**Fig. 2:**
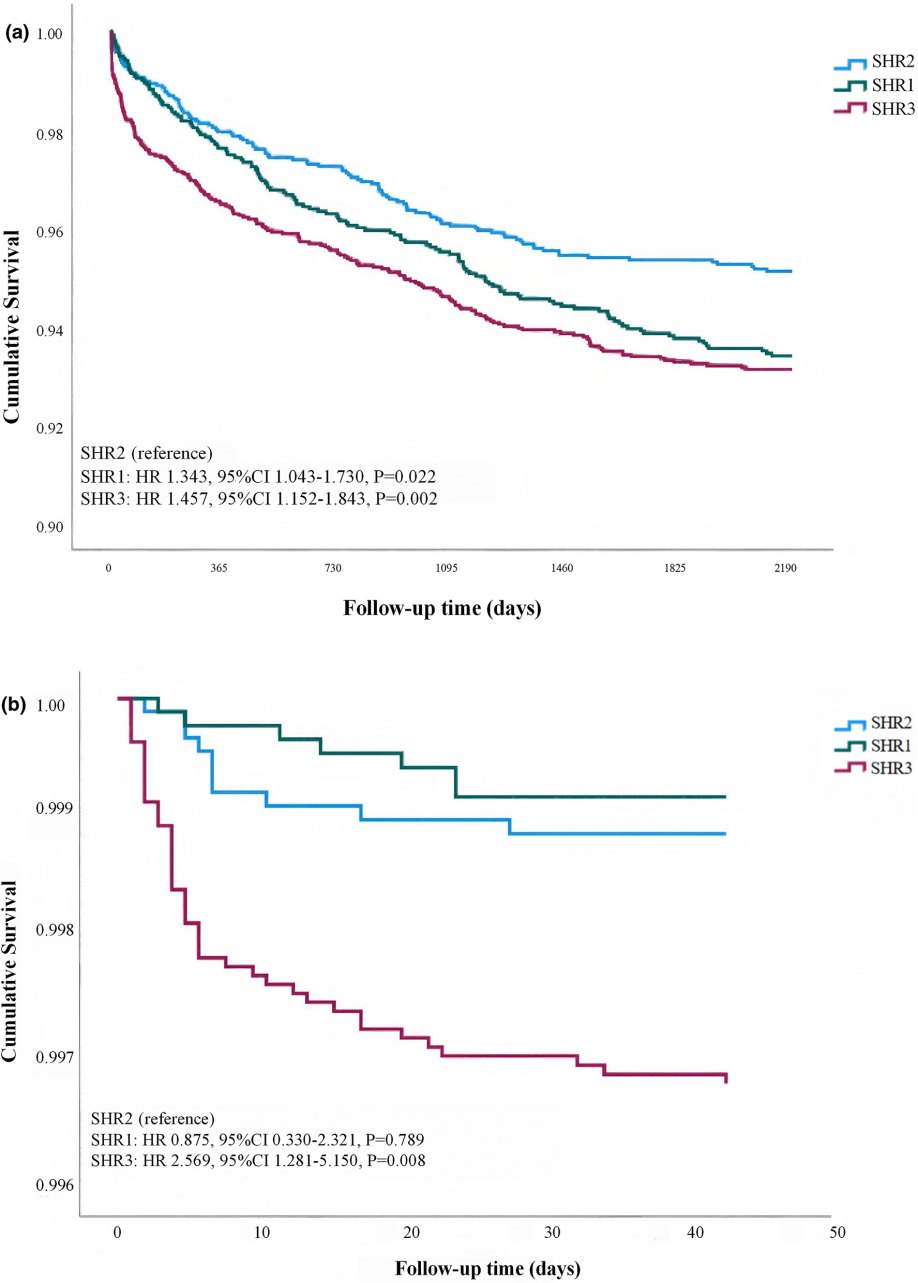
**a** Kaplan-Meier curve of 6-year all-cause mortality. **b** Kaplan-Meier curve of in-hospital all-cause mortality

The Kaplan-Meier curves, adjusted for the variables in model 3, demonstrated disparities in mortality rates among the SHR1, SHR2, and SHR3 cohorts at the 6-year follow-up. Relative to the SHR2 cohort, both the SHR1 and SHR3 cohorts exhibited a significantly elevated mortality risk at the 6-year mark (*P* < 0.01). In terms of in-hospital mortality risk, no significant difference was observed between the SHR1 cohort and the SHR2 cohort, whereas the SHR3 cohort showed a notable increase in risk when compared to the SHR2 cohort (Fig. 2a and b).

### The correlation between the SHR and clinical outcomes in IS patients

**Fig. 3:**
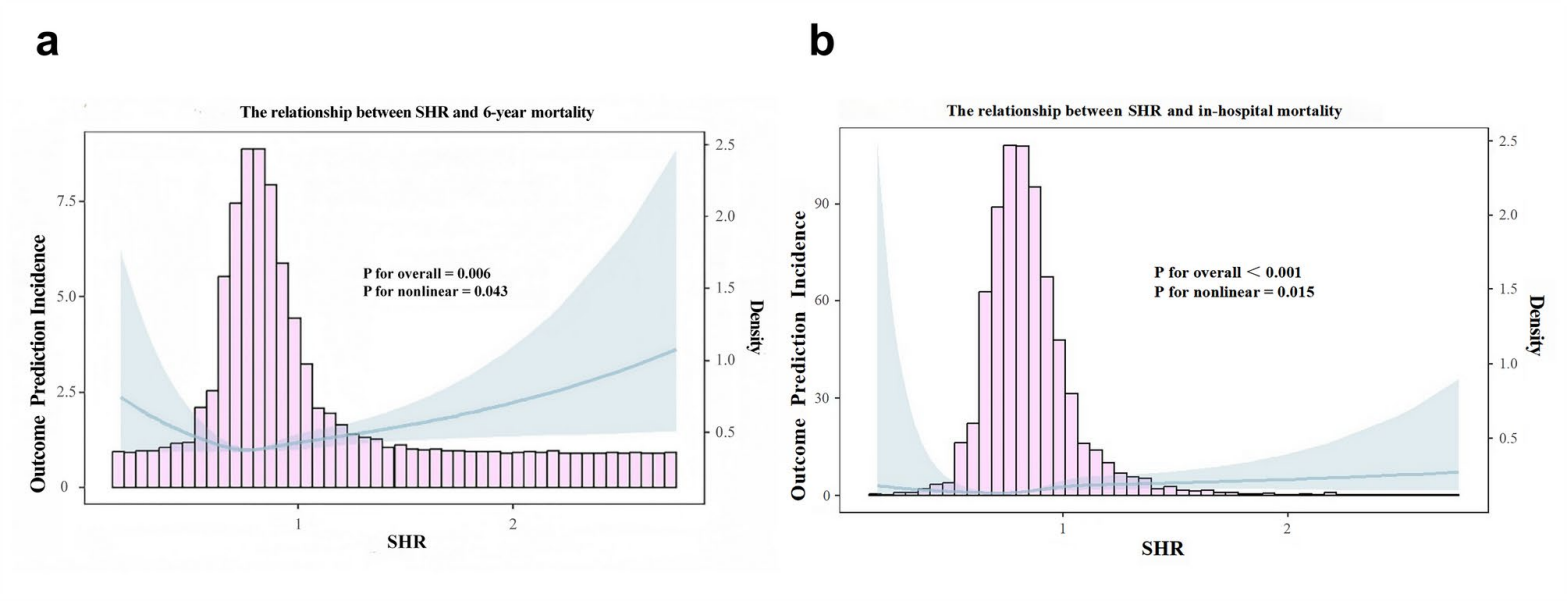
**a** RCS curve of SHR in relation to 6-year all-cause mortality. **b** RCS curve of SHR in relation to in-hospital all-cause mortality

Adjusted factors based on Model 3 in Table 2, the analysis of RCS curves unveiled a nonlinear association between the SHR and six-year all-cause mortality. Notably, the SHR exhibited a U-shaped correlation with six-year mortality (P for overall = 0.001, P for nonlinear = 0.015). A similar nonlinear relationship between the SHR and in-hospital all-cause mortality was also identified; however, the U-shape was absent (refer to Fig. 3a and b).

### Threshold effect analysis of the SHR on mortality in IS patients

To delve deeper into this nonlinear association, a Cox proportional hazards model and a two-piecewise Cox proportional hazards model were employed (*P* < 0.05 for the log-likelihood ratio for both models). The threshold value of the SHR for six-year all-cause mortality was determined to be 0.76. When the SHR surpassed 0.76, the likelihood of mortality over six years from all causes escalated by a factor of 3.877 for each unit increment in the SHR (HR 4.877; 95% CI 3.443–6.907, *P* < 0.001). For in-hospital all-cause mortality, the SHR cut-off point was established at 1.165, and within the range below 1.165, the SHR significantly heightened the risk of in-hospital mortality (HR 31.533; 95% CI 5.571-178.476, *P* < 0.001). Refer to Table 3.

### Stratified analysis

To investigate whether the connection between SHR and all-cause mortality over a six-year span, as well as in-hospital mortality, remains consistent across various conditions, stratified analyses were conducted based on OCSP classification, gender, and age (with 65 years serving as the threshold) [22], HAP, diabetes, AF, dysphagia, and EVT. The adjustment method employed was identical to that utilized in Model 3 in Table 2.

**Fig. 4:**
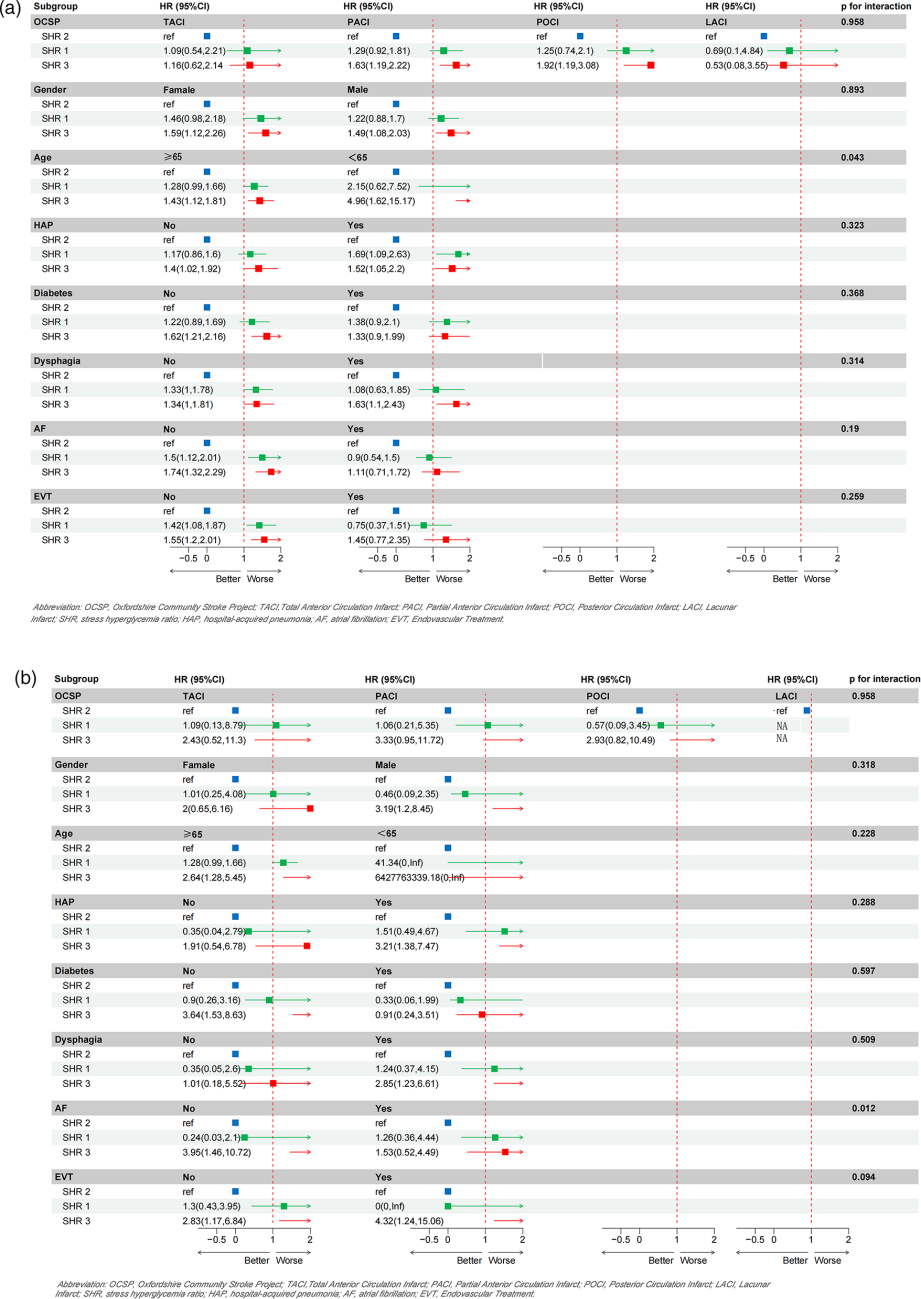
**a** Stratified forest plots of SHR and 6-year all-cause mortality. **b** Stratified forest plots of SHR and in-hospital all-cause mortality

Figure 4a illustrated that, in comparison to the SHR2 group, the six-year mortality risk among the non-diabetic population, non-AF patients, and individuals who had not undergone EVT was markedly elevated in the SHR1 and SHR3 groups, whereas the six-year mortality risk did not demonstrate significance within the diabetic population, AF patients, or those who had received EVT. With the exception of age, other covariates did not exhibit any significant interaction with SHR (*P* = 0.043).

Figure 4b indicated that the SHR3 group presented a heightened risk of in-hospital mortality among males aged 65 and above, those with HAP, non-diabetic individuals, dysphagia, non-AF, regardless of EVT. Aside from AF, other covariates did not reveal significant interaction with SHR (*P* = 0.012).

### The incremental effect of SHR on all-cause mortality

**Fig. 5:**
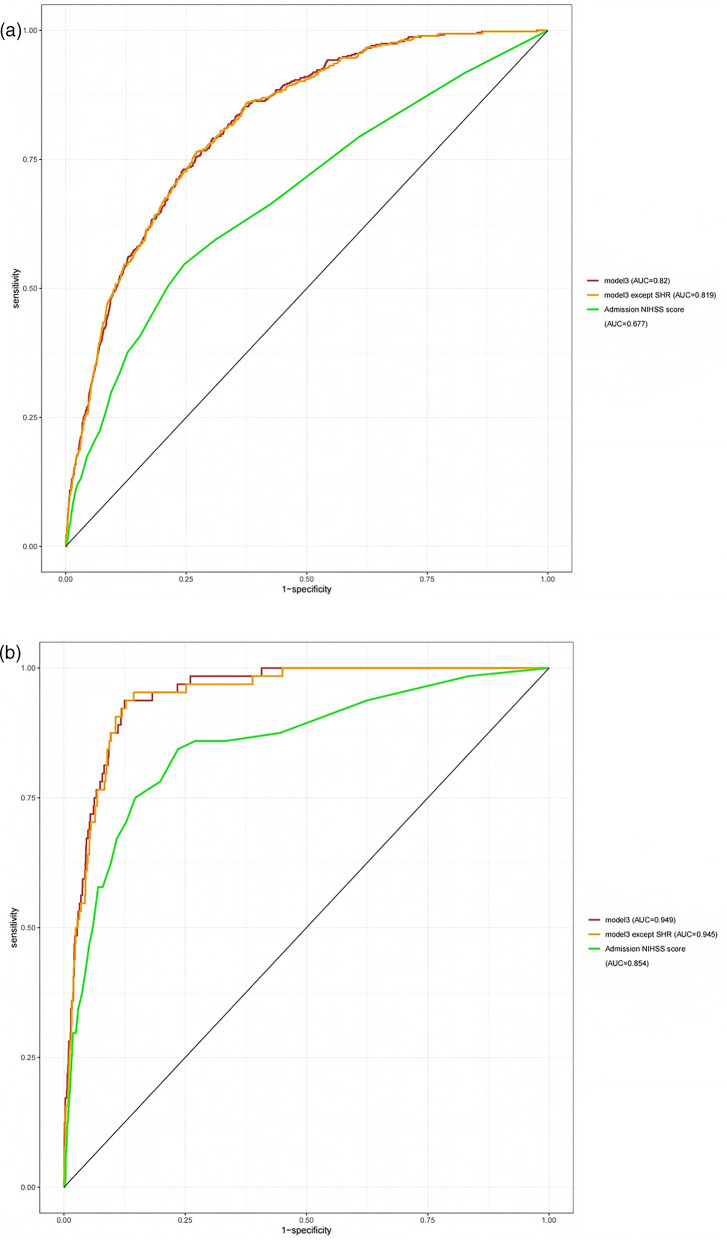
**a** ROC curve analysis of the incremental effect of SHR on 6-year all-cause mortality. **b** ROC curve analysis of the incremental effect of SHR on in-hospital all-cause mortality Supplement

The prognostic significance and additional impact of SHR were evaluated by analyzing the effect of integrating SHR into pre-existing scoring systems on the prediction of both 6-year and in-hospital mortality rates through the construction of the ROC curve and the calculation of the AUC. The findings indicated that the integration of SHR enhanced the predictive precision for mortality rates at both the 6-year mark and during hospitalization. The AUC of the admission NHISS score, Model 3 (in Table 2) without SHR, and Model 3 (in Table 2) concerning 6-year mortality (rising from 0.677 to 0.819 to 0.820), as well as for in-hospital mortality (rising from 0.854 to 0.945 to 0.949), is illustrated in Fig. 5a and b. However, there was no statistically significant difference in the enhancement of AUC after the inclusion of SHR in Model 3 (in Table 2), which did not initially incorporate SHR.

## Discussion

Our investigation has uncovered, for the first time, a distinct correlation between SHR and long-term mortality in patients with IS. A U-shaped nonlinear association was identified between SHR and six-year mortality, revealing a positive correlation when SHR exceeded 0.76. Conversely, the relationship between SHR and long-term mortality was not statistically significant when SHR fell below 0.76. Antiplatelet therapy and statin treatment emerged as protective factors enhancing the long-term survival of IS patients [23, 24], a conclusion further corroborated by our study. Even after adjusting for these variables, SHR persisted as an independent predictor of the risk of long-term mortality. A systematic review and dose-response meta-analysis of a cohort study showed a “J-shaped” nonlinear dose-response relationship between 3-month and 1-year death outcomes and SHR [17]. The risk of 3-month mortality was kept at a relative low point when SHR < 1.5. When SHR > 1.5, the risk of mortality significantly increases. For 1-year mortality, the risk of mortality was kept at a relative low point when SHR < 1.0. When SHR > 1.0, the risk of mortality significantly increases. Our investigation indicated a nonlinear relationship between the SHR and 6-year mortality, characterized by a U-type correlation rather than a J-type. This discrepancy may stem from differences in sample sizes and follow-up durations. Our research additionally indicated that SHR serves as an independent risk factor for in-hospital mortality, revealing a nonlinear correlation between SHR and the likelihood of mortality within hospital settings, thereby reinforcing findings from earlier studies [16, 17, 18]. The threshold for SHR was determined to be 1.165, below which the SHR markedly increased the risk.

Abnormal glucose metabolism and stress-related hyperglycemia are prevalent among critically ill patients. This type of hyperglycemia signifies a physiological response to stress [25], potentially stemming from disorders in glucose metabolism as well as acquired insulin resistance linked to inflammation and organ dysfunction [26]. ABG test provides a swift assessment of blood glucose levels upon admission; however, since it fails to reflect chronic blood glucose variations, it cannot offer a thorough evaluation of glucose levels by itself. Consequently, the SHR has been incorporated into a broader spectrum of medical investigations, potentially serving as a more effective biomarker for severe illnesses after adjusting for baseline blood glucose [14]. SHR has emerged as a possible predictor of adverse outcomes in IS patients [15, 27]. Research conducted by Roberts G et al. demonstrated that the SHR affords the most accurate prognostic insight when examining the correlation between stress-induced hyperglycemia and IS outcomes at the time of admission [13]. Moreover, the SHR can be utilized to forecast long-term clinical outcomes; for instance, it is independently linked to overall and cardiovascular mortality in individuals with Metabolic Syndrome and exhibits a U-shaped association with clinical endpoints [28]. Additionally, the SHR correlates with all-cause and cardiovascular mortality in patients with diabetes or prediabetes, indicating its potential predictive value for these populations [29]. Notably, the SHR is also related to the prognosis of IS patients and may serve as a significant clinical prognostic indicator, particularly regarding the long-term mortality of IS patients. In our research, the RCS analysis revealed a U-shaped relationship between the SHR and long-term mortality in IS patients.

### Association and mechanism between SHR and IS

The relationship and underlying mechanisms between SHR and the severity and mortality of IS patients remain inadequately explored at this time. IS is characterized by dysfunction in cerebral blood perfusion. The link between SHR and adverse outcomes in IS patients may be elucidated by its influence on cerebral blood flow. Elevated glucose levels, through the stimulation of thromboxane A2 release, create an imbalance between vasoactive and procoagulant substances, leading to diminished blood flow [30]. IS patients with hyperglycaemia have reduced plasma fibrinolytic activity and increased levels of fibrinogen activator inhibition, which may reduce vascular recanalization [31]. Furthermore, hyperglycemia fosters oxidative stress and inflammation, driving the production of reactive species and free radicals, which contribute to ischemic cell death linked to hemidesmosomes and aggravate brain edema [32], ultimately impairing the reperfusion of cerebral tissue and resulting in a poorer prognosis for patients [33]. Research using rat stroke models has demonstrated that hyperglycemia initiates the thromboinflammatory cascade and intensifies downstream microvascular thromboinflammation resulting from vascular occlusion. This sequence of events can worsen microcirculatory dysfunction following blood flow restoration, leading to insufficient improvements in brain tissue perfusion (FR) [34]. Previous investigations have also indicated that stress-induced hyperglycemia adversely affects platelet function in acute coronary syndrome patients, potentially causing abnormal platelet aggregation, which may result in microthrombosis and microcirculatory disorders [35]. Acute IS patients necessitate endovascular therapy (EVT) for vascular recanalization; however, delays in this process can provoke secondary reperfusion injury [36, 37], with hyperglycemia exacerbating this condition along with inflammatory aggregation through the promotion of oxidative stress responses [38]. When IS patients present with elevated stress hyperglycemia levels, a heightened inflammatory response to acute critical illness may advance neuroinflammation and the release of neurotoxins and vasoconstrictive factors, further compromising endothelial cells and diminishing vascular repair and protection, thus increasing the risk of intracerebral hemorrhage potentially induced by vascular treatment [39]. Lastly, hyperglycemia enlarges the infarct core in stroke patients, and in animal stroke models, hyperglycemia hastened the onset of cellular acidosis symptoms in the ischemic penumbra, resulting in a greater infarct size compared to hypoglycemic animals treated with insulin. This mechanism may be associated with intracellular acidosis and apoptosis precipitated by cerebral ischemia and hypoxia [40].

### Association between the SHR and all-cause mortality and the underlying mechanism

Our research has unveiled a U-shaped correlation between the SHR and six-year all-cause mortality. Prior investigations have substantiated this U-shaped connection, including the relationship between SHR and both all-cause and cardiovascular mortality among individuals with diabetes or pre-diabetes [29], as well as the association between SHR and short-term and long-term mortality in critically ill patients [41]. Similar findings have also been documented in patients suffering from AF and heart failure [42, 43]. The underlying mechanisms remain ambiguous and may be linked to a variety of factors. Stress hyperglycemia is regarded as a physiological reaction to severe stress, aimed at reestablishing homeostasis. Some research has even suggested that mild to moderate stress hyperglycemia may serve as a protective mechanism during stressful events, particularly in the context of ischemia [44]. This phenomenon has been illustrated through observations made in animal models of hemorrhagic shock, wherein the administration of hypertonic glucose solutions enhances cardiac output, blood pressure, and survival rates [45]. Furthermore, stress hyperglycemia can elevate the expression of cell survival factors, such as vascular endothelial growth factor and hypoxia-inducible factor-1α. Consequently, this leads to diminished apoptosis, reduced infarct size, and enhanced myocardial contractile function, as corroborated in a myocardial infarction rat model [46]. Remarkably, moderate stress hyperglycemia, defined by blood glucose levels ranging from 140 to 220 mg/dL, optimizes cellular glucose uptake while circumventing hypertonicity [47].

Previous studies have indicated that strategies aimed at lowing blood glucose levels during hospitalization may improve outcomes for IS patients with Diabetes Mellitus [48]. Furthermore, achieving glycemic control (normalizing blood glucose to below 130 mg/dL) was correlated with a staggering 4.6-fold reduction in mortality risk when compared to patients experiencing persistent hyperglycemia (*p* < 0.001). This suggests that the normalization of blood glucose within the initial 48 h of hospitalization appears to provide a significant survival advantage for individuals with thromboembolic stroke [49]. Integrating our findings, this implies that implementing suitable hypoglycemic strategies based on SHR may diminish the long-term mortality rates among IS patients. We underscore the necessity for personalized treatment strategies informed by SHR metrics. Concurrently, while we advocate for a robust treatment regimen, it is vital to weigh this against the potential risks of adverse effects, such as hypoglycemic events.

### Prognostic variations among subgroups classified within different SHR groups

SHR layering profoundly influences the long-term outlook for the IS population. To facilitate better clinical identification of high-risk patients, we further stratified the IS population. Our subgroup analysis revealed a noteworthy interaction between SHR and long-term survival within the age subgroup (*P* = 0.043). Elevated SHR significantly impacted the long-term mortality risk for both IS patients aged < 65 years and those aged ≥ 65 years (*P* < 0.05), with a more pronounced effect observed in the < 65 years group (HR = 4.96). Currently, the mechanisms underlying the relationship between age stratification and SHR remain ambiguous; however, several factors may be implicated: (1) Patients younger than 65 are less predisposed to chronic metabolic diseases [50], indicating that an increase in SHR serves as a more direct reflection of acute stress severity. (2) Elevated blood sugar levels impair the phagocytic capacity of white blood cells, disrupting antibody synthesis and compromising immune response [51]. The persistence of inflammation may interact with hyperglycemia, thereby influencing the long-term prognosis for patients. We also discovered that both high SHR and low SHR were associated with significantly elevated long-term mortality in HAP compared to moderate SHR, despite the incidence of HAP being considerably greater in the SHR3 group than in the SHR2 and SHR1 groups (28.39 vs. 15.32 vs. 11.99). Moreover, this study indicated that, in comparison to moderate SHR, long-term mortality significantly increased in the non-AF group and the no EVT group with low SHR or high SHR. This could be attributed to the fact that patients with AF typically receive anticoagulant therapy, which mitigates the risk of thrombosis and subsequent strokes, whereas IS patients without AF lack this protective measure, thereby magnifying the impact of SHR on them. In this study, the anticoagulant utilization rates in the non-AF group and the AF group were 1.2% (47/3794) versus 12.7% (68/536). According to a meta-analysis, EVT was associated with a higher rate of mRS scores of 0 to 2 and reduced mortality across multiple randomized clinical trials [52]. Another investigation indicated that early recanalization served as an independent predictor of favorable clinical outcomes solely in those patients presenting with severe ischemic strokes (NIHSS score > 15; *P* = 0.017). This phenomenon may be linked to EVT’s ability to swiftly restore cerebral blood flow and minimize brain tissue damage, thereby enhancing patient prognosis [53]. We also noted that the correlation between SHR and long-term mortality in IS patients was more pronounced among non-diabetic individuals. This further corroborates the findings from Chen Y and Miao L [54, 55], as elevated blood glucose levels exert varying degrees of detrimental effects on the nervous, cardiovascular, and immune systems [56]. This occurrence may be due to the fact that in individuals without diabetes, a sudden short-term spike or fluctuation in blood glucose levels can significantly exacerbate oxidative stress and lead to endothelial dysfunction [57]. In contrast, diabetic patients, who endure chronic hyperglycemia, may have developed diminished sensitivity to such acute glucose variations. Based on the aforementioned findings, both low SHR and high SHR exert a significant adverse impact on the long-term prognosis of IS patients across different strata, suggesting that blood glucose levels should be maintained at a moderate level for long-term optimal patient care. The outcome was found in the hospital during follow-up. SHR3 group presented a heightened risk of in-hospital mortality among males aged 65 and above, those with HAP, non-diabetic individuals, dysphagia, non-AF, regardless of EVT.

### Limitations

Our research is subject to several limitations. Firstly, it was conducted at a single center; despite adjustments for various variables and additional subgroup analyses, potential confounding factors may still influence the outcomes of this study. Secondly, considering that our sample population is drawn from Shanghai, China, the observed incidence of IS and the differing demographic characteristics suggest that the findings of this retrospective single-center study may not be applicable to other populations. Further investigations are warranted to validate these results. Thirdly, while mechanical thrombectomy techniques have significantly enhanced clinical outcomes in IS cases, only 1.5% of patients in our study underwent this procedure. Such a minor percentage could potentially affect the findings, highlighting the need for further research to affirm the conclusions. Fourthly, our research primarily focused on the correlation between elevated SHR and mortality rates among IS patients. The findings reveal a U-shaped association between SHR and the long-term survival of individuals affected by IS. However, it is crucial to conduct further inquiries to identify the optimal SHR regulation that would provide maximum advantages for patients. Implementing repeated SHR evaluations could offer deeper insights into these results. Additionally, while we did not specifically analyze the influence of glucose regulation on mortality risk, we acknowledge its potential importance. Future studies should comprehensively explore how SHR-focused blood glucose management affects the long-term outcomes for those suffering from IS. Lastly, our findings lend further credence to these assertions, Antiplatelet therapy and statin treatment emerged as protective factors enhancing the survival of IS patients [23, 24]. However, it is important to recognize that while our analysis revealed that SHR remained associated with six-year mortality after accounting for pertinent factors, out-of-hospital treatments may also influence long-term survival outcomes. The potential impact of this on the conclusions drawn from our study necessitates further investigation.

## Conclusions

This research indicates that in individuals with IS, higher levels of SHR are associated with an increased risk of all-cause mortality, observed both at six-year follow-up and during hospitalization. A U-shaped nonlinear correlation exists between SHR and six-year all-cause mortality. This implies that SHR could be a valuable prognostic indicator for adverse long-term outcomes in patients with IS, potentially aiding clinicians in their decision-making processes and risk evaluations.

## Electronic supplementary material

Below is the link to the electronic supplementary material.

Supplementary Material 1

Supplementary Material 2

Supplementary Material 3

Supplementary Material 4

## Data Availability

The data supporting the findings of this study are available from an undisclosed internal stroke database curated by our hospital. This database was established at 2013, originating from the “Shanghai Stroke and Treatment Service System” and conforming to the quality control protocols set forth by the neurology department. Access to this data is limited, as it was utilized under a license for the current study and, therefore, is not publicly accessible. However, the data are available from the corresponding author upon reasonable request.
