# Supplementary material for: Association between stress hyperglycemia ratio (SHR) and long-term mortality in patients with ischemic stroke: a retrospective cohort study
Source: Cardiovasc Diabetol. 2025 Apr 25;24:180. doi: 10.1186/s12933-025-02730-8 (PMC12023360; doi:10.1186/s12933-025-02730-8)
Supplement: Supplementary file 1 — Supplementary Material 1 [file 12933_2025_2730_MOESM1_ESM.doc]

Supplement Table 1 Baseline characteristics of two follow-up endpoints in ischemic stroke patients

| Factors | Total  (n = 4330) | follow-up period for six years | | |  | follow-up period for hospitalization | | |
| --- | --- | --- | --- | --- | --- | --- | --- | --- |
|  |  | Survivor  (n = 3849) | Death  (n = 481) | P Value |  | Survivor  (n = 4266) | Death  (n =64 ) | P Value |
| **Demographic data** |  |  |  |  |  |  |  |  |
| Male [n (%)] | 2805 (64.78) | 2532 (65.78) | 273 (56.76) | <0.001 |  | 2774 (65.03) | 31 (48.44) | 0.006 |
| Age (Mean ± SD, years) | 69.28 ± 13.41 | 67.96 ± 13.28 | 79.84 ± 9.12 | <0.001 |  | 69.10 ± 13.37 | 81.77 ± 9.86 | <0.001 |
| BMI (Mean ± SD) | 24.46 ± 2.98 | 24.48 ± 2.97 | 24.33 ± 3.06 | 0.293 |  | 24.47 ± 2.97 | 23.95 ± 3.37 | 0.167 |
| Waist circumference (Mean±SD, cm) | 84.18 ± 8.31 | 84.27 ± 8.34 | 83.45 ± 8.07 | 0.046 |  | 84.19 ± 8.29 | 83.71 ± 9.64 | 0.653 |
| **OCSP [n (%)]** |  |  |  | <0.001 |  |  |  | <0.001 |
| TACI [n (%)] | 421 (9.72) | 349 (9.07) | 72 (14.97) |  |  | 401 (9.40) | 20 (31.25) |  |
| PACI [n (%)] | 2605 (60.16) | 2330 (60.54) | 275 (57.17) |  |  | 2580 (60.48) | 25 (39.06) |  |
| POCI [n (%)] | 1110 (25.64) | 988 (25.67) | 122 (25.36) |  |  | 1091 (25.57) | 19 (29.69) |  |
| LACI [n (%)] | 194 (4.48) | 182 (4.73) | 12 (2.49) |  |  | 194 (4.55) | 0 (0.00) |  |
| **TOAST [n (%)]** |  |  |  | <0.001 |  |  |  | <0.001 |
| Large-artery atherosclerosis [n (%)] | 1686 (38.94) | 1455 (37.80) | 231 (48.02) |  |  | 1654 (38.77) | 32 (50.00) |  |
| Cardio embolism [n (%)] | 473 (10.92) | 364 (9.46) | 109 (22.66) |  |  | 446 (10.45) | 27 (42.19) |  |
| Small-vessel occlusion [n (%)] | 2025 (46.77) | 1892 (49.16) | 133 (27.65) |  |  | 2022 (47.40) | 3 (4.69) |  |
| Stroke of other determined etiology [n (%)] | 55 (1.27) | 52 (1.35) | 3 (0.62) |  |  | 54 (1.27) | 1 (1.56) |  |
| Stroke of undetermined etiology [n (%)] | 91 (2.10) | 86 (2.23) | 5 (1.04) |  |  | 90 (2.11) | 1 (1.56) |  |
| **Smoke [n (%)]** |  |  |  | 0.100 |  |  |  | 0.798 |
| Never smoked [n (%)] | 3074 (70.99) | 2720 (70.67) | 354 (73.60) |  |  | 3027 (70.96) | 47 (73.44) |  |
| Used to smoke [n (%)] | 208 (4.80) | 180 (4.68) | 28 (5.82) |  |  | 206 (4.83) | 2 (3.12) |  |
| Still smoking [n (%)] | 1048 (24.20) | 949 (24.66) | 99 (20.58) |  |  | 1033 (24.21) | 15 (23.44) |  |
| Stroke onset to hospitalization [M (25%,75%), hours] | 8.0 (2.1, 30.0) | 8.3 (2.1, 31.0) | 5.5 (1.6, 24.0) | <0.001 |  | 8.1 (2.1, 30.2) | 2.1 (1.0, 6.0) | <0.001 |
| **Past medical history [n (%)]** |  |  |  |  |  |  |  |  |
| Prior cerebral infarction [n (%)] | 797 (18.41) | 659 (17.12) | 138 (28.69) | <0.001 |  | 778 (18.24) | 19 (29.69) | 0.019 |
| Transient ischemic attack [n (%)] | 20 (0.46) | 18 (0.47) | 2 (0.42) | 1.000 |  | 19 (0.45) | 1 (1.56) | 0.258 |
| Myocardial infarction [n (%)] | 26 (0.60) | 26 (0.68) | 0 (0.00) | 0.135 |  | 26 (0.61) | 0 (0.00) | 1.000 |
| Hypertension [n (%)] | 3110 (71.82) | 2722 (70.72) | 388 (80.67) | <0.001 |  | 3060 (71.73) | 50 (78.12) | 0.259 |
| Diabetes mellitus [n (%)] | 1314 (30.35) | 1127 (29.28) | 187 (38.88) | <0.001 |  | 1292 (30.29) | 22 (34.38) | 0.48 |
| Atrial fibrillation [n (%)] | 536 (12.38) | 410 (10.65) | 126 (26.20) | <0.001 |  | 503 (11.79) | 33 (51.56) | <0.001 |
| Hyperlipidemia [n (%)] | 27 (0.62) | 26 (0.68) | 1 (0.21) | 0.426 |  | 27 (0.63) | 0 (0.00) | 1.000 |
| Cerebral hemorrhage [n (%)] | 104 (2.40) | 95 (2.47) | 9 (1.87) | 0.42 |  | 104 (2.44) | 0 (0.00) | 0.394 |
| Dementia [n (%)] | 41 (0.95) | 32 (0.83) | 9 (1.87) | 0.049 |  | 39 (0.91) | 2 (3.12) | 0.122 |
| Mental health disorder [n (%)] | 21 (0.48) | 20 (0.52) | 1 (0.21) | 0.562 |  | 21 (0.49) | 0 (0.00) | 1.000 |
| Chronic obstructive Pulmonary disease [n (%)] | 72 (1.66) | 60 (1.56) | 12 (2.49) | 0.13 |  | 71 (1.66) | 1 (1.56) | 1.000 |
| Spontaneous intracerebral hemorrhage [n (%)] | 54 (1.25) | 44 (1.14) | 10 (2.08) | 0.081 |  | 52 (1.22) | 2 (3.12) | 0.189 |
| Family history of stroke [n (%)] | 15 (0.35) | 13 (0.34) | 2 (0.42) | 1.000 |  | 15 (0.35) | 0 (0.00) | 1.000 |
| Heart valve replacement surgery [n (%)] | 7 (0.16) | 7 (0.18) | 0 (0.00) | 1.000 |  | 7 (0.16) | 0 (0.00) | 1.000 |
| **The severity of the disease** |  |  |  |  |  |  |  |  |
| Pre-morbidity mRS score [M (IQR)] | 2.00 (1.00, 2.00) | 2.00 (1.00, 2.00) | 2.00 (1.00, 2.00) | 0.003 |  | 2.00 (1.00, 2.00) | 2.00 (1.00, 3.00) | 0.077 |
| Admission NIHSS score [M (IQR)] | 2.00 (1.00, 5.00) | 2.00 (1.00, 4.00) | 6.00 (2.00, 12.00) | <0.001 |  | 2.00 (1.00, 5.00) | 14.50 (8.75, 21.00) | <0.001 |
| Dysphagia [n (%)] | 489 (11.44) | 317 (8.36) | 172 (35.91) | <0.001 |  | 433 (10.29) | 56 (87.50) | <0.001 |
| MAP (Mean ± SD, mmHg) | 105.94 ± 14.25 | 106.09 ± 14.17 | 104.76 ± 14.82 | 0.062 |  | 105.96 ± 14.20 | 104.96 ± 16.97 | 0.642 |
| Pulse (Mean ± SD, Times/minute) | 77.76 ± 14.36 | 77.41 ± 13.70 | 80.56 ± 18.59 | <0.001 |  | 77.60 ± 14.07 | 88.16 ± 25.28 | 0.001 |
| **Treatment methods:** |  |  |  |  |  |  |  |  |
| Hypoglycemic agents [n (%)] | 1477 (34.11) | 1303 (33.85) | 174 (36.17) | 0.311 |  | 1477 (34.62) | 0 (0.00) | <0.001 |
| Antiplatelet drug therapy [n (%)] | 4163 (96.14) | 3736 (97.06) | 427 (88.77) | <0.001 |  | 4113 (96.41) | 50 (78.12) | <0.001 |
| anticoagulant drugs [n (%)] | 115 (2.66) | 101 (2.62) | 14 (2.91) | 0.713 |  | 114 (2.67) | 1 (1.56) | 0.876 |
| antihypertensive medications [n (%)] | 3315 (76.56) | 2971 (77.19) | 344 (71.52) | 0.006 |  | 3315 (77.71) | 0 (0.00) | <0.001 |
| lipid-lowering treatments [n (%)] | 4131 (95.40) | 3731 (96.93) | 400 (83.16) | <0.001 |  | 4130 (96.81) | 1 (1.56) | <0.001 |
| Endovascular Treatment [n (%)] | 671 (15.50) | 597 (15.51) | 74 (15.38) | 0.943 |  | 653 (15.31) | 18 (28.12) | 0.005 |
| Intravenous thrombolysis utilizing alteplase [n (%)] | 650 (15.01) | 576 (14.96) | 74 (15.38) | 0.808 |  | 632 (14.81) | 18 (28.12) | 0.003 |
| Mechanical thrombectomy [n (%)] | 62 (1.50) | 53 (1.44) | 9 (1.97) | 0.379 |  | 59 (1.45) | 3 (4.76) | 0.068 |
| **Laboratory indicators** |  |  |  |  |  |  |  |  |
| LDL (M (IQR), mmol/L) | 2.66 (2.05, 3.30) | 2.67 (2.07, 3.31) | 2.47 (1.87, 3.20) | <0.001 |  | 2.66 (2.05, 3.31) | 2.53 (1.93, 3.12) | 0.239 |
| Hcy [M (IQR), umol/L] | 14.80 (11.40, 20.20) | 14.60 (11.30, 19.90) | 16.90 (12.65, 22.85) | <0.001 |  | 14.80 (11.40, 20.10) | 18.20 (12.55, 24.30) | 0.019 |
| HbA1c (Mean±SD) | 6.67 ± 1.73 | 6.65 ± 1.72 | 6.82 ± 1.79 | 0.037 |  | 6.66 ± 1.73 | 6.78 ± 1.97 | 0.584 |
| ABG (Mean±SD, mmol/L) | 6.70 ± 2.69 | 6.62 ± 2.61 | 7.36 ± 3.20 | <0.001 |  | 6.67 ± 2.65 | 8.67 ± 4.22 | <0.001 |
| SCr [M (IQR), umol/L] | 72.75 (61.61, 86.29) | 71.99 (61.42, 85.20) | 79.00 (64.03, 95.52) | <0.001 |  | 72.50 (61.59, 86.03) | 84.01 (73.91, 106.07) | <0.001 |
| BUN [M (IQR), mmol/L] | 5.10 (4.20, 6.20) | 5.00 (4.10, 6.10) | 5.70 (4.60, 7.50) | <0.001 |  | 5.10 (4.20, 6.20) | 6.85 (5.68, 9.06) | <0.001 |
| UA (Mean ± SD, umol/L) | 330.47 ± 102.11 | 330.50 ± 101.21 | 330.16 ± 109.15 | 0.947 |  | 330.03 ± 101.66 | 359.66 ± 126.75 | 0.07 |
| INR (Mean±SD) | 0.95 ± 0.15 | 0.95 ± 0.15 | 0.99 ± 0.17 | <0.001 |  | 0.95 ± 0.15 | 1.03 ± 0.13 | <0.001 |
| SHR (Mean±SD) | 0.85 ± 0.20 | 0.84 ± 0.19 | 0.91 ± 0.27 | <0.001 |  | 0.84 ± 0.19 | 1.07 ± 0.36 | <0.001 |
| **Outcomes** |  |  |  |  |  |  |  |  |
| Length of hospitalization [M (IQR), days] | 10.00 (8.00, 13.00) | 10.00 (8.00, 13.00) | 12.00 (9.00, 18.00) | <0.001 |  | 10.00 (8.00, 13.00) | 5.00 (3.00, 13.25) | <0.001 |
| Total hospitalization expenses [M (IQR), Thousand yuan] | 14.7 (11.3, 19.9) | 14.5(11.2, 19.3) | 17.7 (12.4, 27.7) | <0.001 |  | 14.7 (11.4, 19.8) | 14.9 (9.7, 3.2) | 0.619 |
| Total hospitalization drug expenses [M (IQR), Thousand yuan] | 7.1(4.5, 10.4) | 6.9(4.4, 10.2) | 8.8(5.6, 14.1) | <0.001 |  | 7.1(4.5, 10.4) | 7.0 (3.4, 14.1) | 0.971 |
| HAP Incidence [n (%)] | 804 (18.57) | 587 (15.25) | 217 (45.11) | <0.001 |  | 750 (17.58) | 54 (84.38) | <0.001 |
| SHR (tertiles) |  |  |  | <0.001 |  |  |  | <0.001 |
| SHR1 group [n (%)] | 1443 (33.33) | 1300 (33.78) | 143 (29.73) |  |  | 1436 (33.66) | 7 (10.94) |  |
| SHR2 group [n (%)] | 1443 (33.33) | 1325 (34.42) | 118 (24.53) |  |  | 1433 (33.59) | 10 (15.62) |  |
| SHR3 group [n (%)] | 1444 (33.35) | 1224 (31.80) | 220 (45.74) |  |  | 1397 (32.75) | 47 (73.44) |  |

Abbreviation: ABG, admission blood glucose; BMI, body mass index; BUN, blood urea nitrogen; Hcy, homocysteine; HbA1c, glycated hemoglobin; HAP, hospital-acquired pneumonia; INR, international normalized ratio; LACI, lacunar infarct; LDL, low-density lipoprotein; MAP, mean arterial pressure; mRS, modified rankin scale; NIHSS, national institute of health stroke scale; OCSP, oxfordshire community stroke project; POCI, posterior circulation infarct; PACI, partial anterior circulation infarct; SHR, stress hyperglycemia ratio; SCr, serum creatinine; TACI, total anterior circulation infarct; TOAST, trial of org 10172 in acutes troke treatment; UA, uric acid.
