# Supplementary material for: Association between stress hyperglycemia ratio (SHR) and long-term mortality in patients with ischemic stroke: a retrospective cohort study
Source: Cardiovasc Diabetol. 2025 Apr 25;24:180. doi: 10.1186/s12933-025-02730-8 (PMC12023360; doi:10.1186/s12933-025-02730-8)
Supplement: Supplementary file 2 — Supplementary Material 2 [file 12933_2025_2730_MOESM2_ESM.doc]

Supplement Table 2 Univariate Cox regression analysis of six-year and in-hospital mortality

| **Factors** | Follow-up for 6 years | | Follow-up hospitalization period | |
| --- | --- | --- | --- | --- |
|  | HR (95% CI) | P Value | HR (95% CI) | P Value |
| **Demographic data** |  |  |  |  |
| Male | 0.696 (0.581 - 0.834) | < 0.001 | 0.509 (0.311 ~ 0.830) | 0.007 |
| Age | 1.085 (1.075 - 1.094) | < 0.001 | 1.101 (1.073 ~ 1.130) | <0.001 |
| BMI | 0.983 (0.954 - 1.013) | 0.266 | 0.943 (0.867 ~ 1.025) | 0.167 |
| Waist circumference | 0.988 (0.977 - 1.000) | 0.043 | 0.993 (0.963 ~ 1.024) | 0.657 |
| **OCSP** |  |  |  |  |
| TACI | Reference |  | Reference |  |
| PAC | 0.582 (0.449 - 0.754) | < 0.001 | 0.197 (0.109 ~ 0.354) | <0.001 |
| POCI | 0.606 (0.453 - 0.811) | < 0.001 | 0.352 (0.188 ~ 0.660) | 0.001 |
| LACI | 0.331 (0.180 - 0.610) | < 0.001 | 0.000 (0.000 ~ Inf) | 0.994 |
| **TOAST** |  |  |  |  |
| Large-artery atherosclerosis | Reference |  | Reference |  |
| Cardio embolism | 1.826 (1.454 - 2.293) | < 0.001 | 3.070 (1.840 ~ 5.124) | <0.001 |
| Small-vessel occlusion | 0.459 (0.371 - 0.568) | < 0.001 | 0.077 (0.024 ~ 0.253) | <0.001 |
| Stroke of other determined etiology | 0.383 (0.123 - 1.198) | 0.099 | 0.961 (0.131 ~ 7.030) | 0.968 |
| Stroke of undetermined etiology | 0.388 (0.160 - 0.941) | 0.036 | 0.578 (0.079 ~ 4.234) | 0.59 |
| **Smoke** |  |  |  |  |
| Never smoked | Reference |  | Reference |  |
| Used to smoke | 1.174 (0.799 - 1.725) | 0.414 | 0.625 (0.152 ~ 2.575) | 0.516 |
| Still smoking | 0.816 (0.653 - 1.019) | 0.073 | 0.937 (0.524 ~ 1.675) | 0.826 |
| Stroke onset to hospitalization | 1.000 (1.000 - 1.000) | 0.116 | 0.999 (0.999 ~ 0.999) | 0.009 |
| **Past medical history** |  |  |  |  |
| Prior cerebral infarction | 1.852 (1.520 - 2.257) | < 0.001 | 1.881 (1.100 ~ 3.216) | 0.021 |
| Transient ischemic attack | 0.901 (0.225 - 3.615) | 0.884 | 3.492 (0.484 ~ 25.179) | 0.215 |
| Myocardial infarction | 0.000 (0.000 - Inf) | 0.988 | 0.000 (0.000 ~ Inf) | 0.996 |
| Hypertension | 1.676 (1.337 - 2.102) | <0.001 | 1.402 (0.775 ~ 2.536) | 0.264 |
| Diabetes mellitus | 1.488 (1.239 - 1.787) | <0.001 | 1.200 (0.717 ~ 2.011) | 0.488 |
| Atrial fibrillation | 2.798 (2.284 - 3.429) | <0.001 | 7.751 (4.747 ~ 12.656) | <0.001 |
| Hyperlipidemia | 0.318 (0.045 - 2.263) | 0.253 | 0.000 (0.000 ~ Inf) | 0.996 |
| Cerebral hemorrhage | 0.764 (0.395 - 1.477) | 0.423 | 0.000 (0.000 ~ Inf) | 0.995 |
| Dementia | 2.137 (1.105 - 4.133) | 0.024 | 3.423 (0.837 ~ 13.992) | 0.087 |
| Mental health disorder | 0.405 (0.057 - 2.882) | 0.367 | 0.000 (0.000 ~ Inf) | 0.994 |
| Chronic obstructive Pulmonary disease | 1.550 (0.874 - 2.750) | 0.134 | 0.942 (0.131 ~ 6.789) | 0.952 |
| Spontaneous intracerebral hemorrhage | 1.733 (0.926 - 3.242) | 0.085 | 2.566 (0.628 ~ 10.492) | 0.19 |
| Family history of stroke | 1.147 (0.286 - 4.599) | 0.847 | 0.000 (0.000 ~ Inf) | 0.995 |
| Heart valve replacement surgery | 0.000 (0.000 - Inf) | 0.986 | 0.000 (0.000 ~ Inf) | 0.995 |
| **The severity of the disease** |  |  |  |  |
| Pre-morbidity mRS score | 1.205 (1.123 - 1.293) | <0.001 | 1.356 (1.143 ~ 1.609) | <0.001 |
| Admission NIHSS score | 1.082 (1.071 - 1.092) | <0.001 | 1.138 (1.117 ~ 1.160) | <0.001 |
| Dysphagia | 5.444 (4.516 - 6.563) | <0.001 | 57.550 (27.434 ~ 120.726) | <0.001 |
| MAP | 0.994 (0.987 - 1.000) | 0.052 | 0.995 (0.978 ~ 1.013) | 0.581 |
| Pulse | 1.014 (1.008 - 1.020) | <0.001 | 1.035 (1.024 ~ 1.047) | <0.001 |
| **Treatment methods:** |  |  |  |  |
| Hypoglycemic agents | 1.080 (0.896 - 1.300) | 0.419 | 0.000 (0.000 ~ Inf) | 0.992 |
| Antiplatelet drug therapy | 0.266 (0.200 - 0.353) | <0.001 | 0.137 (0.076 ~ 0.249) | <0.001 |
| anticoagulant drugs | 1.113 (0.654 - 1.894) | 0.694 | 0.584 (0.081 ~ 4.211) | 0.594 |
| antihypertensive medications | 0.732 (0.601 - 0.892) | 0.002 | 0.000 (0.000 ~ Inf) | 0.994 |
| lipid-lowering treatments | 0.161 (0.127 - 0.205) | < 0.001 | 0.001 (0.000 ~ 0.005) | <0.001 |
| Endovascular Treatment | 1.002 (0.782 – 1.283) | 0.988 | 2.153 (1.249 ~ 3.713) | 0.006 |
| Intravenous thrombolysis utilizing alteplase | 1.043 (0.814 - 1.336) | 0.740 | 2.237 (1.297 ~ 3.857) | 0.004 |
| Mechanical thrombectomy | 1.378 (0.713 - 2.666) | 0.341 | 3.338 (1.047 ~ 10.642) | 0.042 |
| **Laboratory indicators** |  |  |  |  |
| LDL | 0.848 (0.768 - 0.936) | 0.001 | 0.860 (0.656 ~ 1.128) | 0.277 |
| Hcy | 1.009 (1.002 - 1.015) | 0.006 | 1.014 (0.999 ~ 1.030) | 0.058 |
| HbA1c | 1.050 (1.001 - 1.101) | 0.046 | 1.038 (0.908 ~ 1.187) | 0.585 |
| ABG | 1.082 (1.054 - 1.111) | <0.001 | 1.177 (1.113 ~ 1.244) | <0.001 |
| SCr | 1.003 (1.002 - 1.004) | <0.001 | 1.004 (1.002 ~ 1.006) | <0.001 |
| BUN | 1.140 (1.110 - 1.169) | <0.001 | 1.228 (1.166 ~ 1.293) | <0.001 |
| UA | 1.000 (0.999 - 1.001) | 0.958 | 1.003 (1.001 ~ 1.005) | 0.022 |
| INR | 2.077 (1.604 - 2.689) | <0.001 | 2.620 (1.564 ~ 4.389) | <0.001 |
| SHR | 3.690 (2.630 - 5.179) | <0.001 | 7.940 (4.461 - 14.131) | <0.001 |
| **Outcomes** |  |  |  |  |
| Length of hospitalization | 1.051 (1.042 - 1.060) | <0.001 | 0.903(0.846-0.964) 0.002 | 0.002 |
| HAP Incidence | 4.172 (3.486 - 4.994) | <0.001 | 24.505 (12.480 ~ 48.118) | <0.001 |
| **SHR (tertiles)** |  |  |  |  |
| SHR2 group | Reference |  | Reference |  |
| SHR1 group | 1.218 (0.954 - 1.554) | 0.113 | 0.725 (0.276 - 1.905) | 0.514 |
| SHR3 group | 1.959 (1.566 - 2.450) | <0.001 | 3.956 (1.989 - 7.868) | <0.001 |

Abbreviation: ABG, admission blood glucose; BMI, body mass index; BUN, blood urea nitrogen; Hcy, homocysteine; HbA1c, glycated hemoglobin; HAP, hospital-acquired pneumonia; INR, international normalized ratio; LACI, lacunar infarct; LDL, low-density lipoprotein; MAP, mean arterial pressure; mRS, modified rankin scale; NIHSS, national institute of health stroke scale; OCSP, oxfordshire community stroke project; POCI, posterior circulation infarct; PACI, partial anterior circulation infarct; SHR, stress hyperglycemia ratio; SCr, serum creatinine; TACI, total anterior circulation infarct; TOAST, trial of org 10172 in acutes troke treatment; UA, uric acid.
