# Supplementary material for: Association between stress hyperglycemia ratio (SHR) and long-term mortality in patients with ischemic stroke: a retrospective cohort study
Source: Cardiovasc Diabetol. 2025 Apr 25;24:180. doi: 10.1186/s12933-025-02730-8 (PMC12023360; doi:10.1186/s12933-025-02730-8)
Supplement: Supplementary file 3 — Supplementary Material 3 [file 12933_2025_2730_MOESM3_ESM.doc]

Supplement Table3 Multivariate Cox regression analysis of six-year mortality

|  | HR (95% CI) | P Value |
| --- | --- | --- |
| **Model 1** |  |  |
| SHR | 1.642 (1.132 - 2.380) | 0.009 |
| Age | 1.073 (1.062 - 1.085) | < 0.001 |
| Antiplatelet drug therapy | 0.712 (0.522 - 0.970) | 0.031 |
| lipid-lowering treatments | 0.534 (0.387 - 0.736) | < 0.001 |
| Diabetes mellitus | 1.655 (1.359 - 2.015) | < 0.001 |
| Dysphagia | 1.715 (1.284 - 2.289) | < 0.001 |
| Prior cerebral infarction | 1.418 (1.152 - 1.745) | < 0.001 |
| HAP | 1.459 (1.160 - 1.834) | 0.001 |
| **Model 2** |  |  |
| SHR divided into tertiles |  |  |
| SHR2 group | Reference |  |
| SHR1 group | 1.275 (0.996 - 1.633) | 0.054 |
| SHR3 group | 1.417 (1.126 - 1.784) | 0.003 |
| Age | 1.073 (1.061 - 1.084) | < 0.001 |
| Antiplatelet drug therapy | 0.679 (0.498 - 0.926) | 0.014 |
| lipid-lowering treatments | 0.529 (0.383 - 0.730) | < 0.001 |
| Diabetes mellitus | 1.610 (1.322 - 1.962) | < 0.001 |
| Dysphagia | 1.748 (1.309 - 2.333) | < 0.001 |
| Prior cerebral infarction | 1.397 (1.135 - 1.719) | 0.002 |
| HAP | 1.476 (1.172 - 1.858) | < 0.001 |

Model 1: Multivariate Cox analysis: SHR as a continuous variable;

Model 2: Multivariate Cox analysis: SHR as a categorical variable;

Abbreviation: HAP, hospital-acquired pneumonia; SHR, stress hyperglycemia ratio.
