# Supplementary material for: Association between stress hyperglycemia ratio (SHR) and long-term mortality in patients with ischemic stroke: a retrospective cohort study
Source: Cardiovasc Diabetol. 2025 Apr 25;24:180. doi: 10.1186/s12933-025-02730-8 (PMC12023360; doi:10.1186/s12933-025-02730-8)
Supplement: Supplementary file 4 — Supplementary Material 4 [file 12933_2025_2730_MOESM4_ESM.doc]

Supplement Table4 Multivariate Cox regression analysis of in-hospital mortality

|  | HR (95% CI) | P Value |
| --- | --- | --- |
| **Model 1** |  |  |
| SHR | 3.839 (2.013 - 7.319) | < 0.001 |
| Antiplatelet drug therapy | 0.503 (0.268 - 0.943) | 0.032 |
| SCr | 1.004 (1.001 - 1.007) | 0.004 |
| Admission NIHSS score | 1.074 (1.046 - 1.103) | < 0.001 |
| age | 1.062 (1.030 - 1.094) | < 0.001 |
| OCSP |  |  |
| TACI | Reference |  |
| PACI | 0.406 (0.218 - 0.759) | 0.005 |
| POCI | 1.590 (0.801 - 3.159) | 0.185 |
| LACI | 0.000 (0.000 - Inf) | 0.996 |
| TOAST |  |  |
| Large-artery atherosclerosis | Reference |  |
| Cardio embolism | 1.364 (0.795 - 2.340) | 0.260 |
| Small-vessel occlusion | 0.151 (0.045 - 0.502) | 0.002 |
| Stroke of other determined etiology | 0.850 (0.106 - 6.811) | 0.878 |
| Stroke of undetermined etiology | 1.744 (0.233 - 13.042) | 0.588 |
| **Model 2** |  |  |
| SHR divided into tertiles |  |  |
| SHR2 group | Reference |  |
| SHR1 group | 0.946 (0.356 - 2.511) | 0.911 |
| SHR3 group | 2.874 (1.438 - 5.742) | 0.003 |
| Antiplatelet drug therapy | 0.493 (0.266 - 0.914) | 0.025 |
| SCr | 1.004 (1.001 - 1.006) | 0.006 |
| Admission NIHSS score | 1.076 (1.048 - 1.105) | < 0.001 |
| age | | 1.059 (1.028 - 1.091) | < 0.001 | | --- | --- | |  |
| OCSP |  |  |
| TACI | Reference |  |
| PACI | 0.413 (0.221 - 0.771) | 0.006 |
| POCI | 1.481 (0.748 - 2.930) | 0.260 |
| LACI | 0.000 (0.000 - Inf) | 0.996 |
| TOAST |  |  |
| Large-artery atherosclerosis | Reference |  |
| Cardio embolism | 1.346 (0.778 - 2.327) | 0.288 |
| Small-vessel occlusion | 0.153 (0.046 - 0.510) | 0.002 |
| Stroke of other determined etiology | 1.102 (0.138 - 8.795) | 0.927 |
| Stroke of undetermined etiology | 1.599 (0.215 - 11.905) | 0.647 |

Model 1: Multivariate Cox analysis: SHR as a continuous variable;

Model 2: Multivariate Cox analysis: SHR as a categorical variable;

Abbreviation: LACI, lacunar infarct; NIHSS, national institute of health stroke scale; OCSP, oxfordshire community stroke project; POCI, posterior circulation infarct; PACI, partial anterior circulation infarct; SHR, stress hyperglycemia ratio; SCr, serum creatinine; TACI, total anterior circulation infarct; TOAST, trial of org 10172 in Acute stroke treatment.
